# Heat-Related Health Impacts under Scenarios of Climate and Population Change

**DOI:** 10.3390/ijerph15112438

**Published:** 2018-11-01

**Authors:** Philip E. Morefield, Neal Fann, Anne Grambsch, William Raich, Christopher P. Weaver

**Affiliations:** 1 Office of Research and Development, National Center for Environmental Assessment, US Environmental Protection Agency, Washington, DC 20460, USA; grambsch.annee@gmail.com (A.G.); weaver.chris@epa.gov (C.P.W.); 2 Office of Air and Radiation, Office of Air Quality, Planning and Standards, US Environmental Protection Agency, Durham, NC 27709, USA; fann.neal@epa.gov; 3 Industrial Economics, Inc., Cambridge, MA 02140, USA; wraich@indecon.com

**Keywords:** climate change, heat-related mortality, risk assessment

## Abstract

Recent assessments have found that a warming climate, with associated increases in extreme heat events, could profoundly affect human health. This paper describes a new modeling and analysis framework, built around the Benefits Mapping and Analysis Program—Community Edition (BenMAP), for estimating heat-related mortality as a function of changes in key factors that determine the health impacts of extreme heat. This new framework has the flexibility to integrate these factors within health risk assessments, and to sample across the uncertainties in them, to provide a more comprehensive picture of total health risk from climate-driven increases in extreme heat. We illustrate the framework’s potential with an updated set of projected heat-related mortality estimates for the United States. These projections combine downscaled Coupled Modeling Intercomparison Project 5 (CMIP5) climate model simulations for Representative Concentration Pathway (RCP)4.5 and RCP8.5, using the new Locating and Selecting Scenarios Online (LASSO) tool to select the most relevant downscaled climate realizations for the study, with new population projections from EPA’s Integrated Climate and Land Use Scenarios (ICLUS) project. Results suggest that future changes in climate could cause approximately from 3000 to more than 16,000 heat-related deaths nationally on an annual basis. This work demonstrates that uncertainties associated with both future population and future climate strongly influence projected heat-related mortality. This framework can be used to systematically evaluate the sensitivity of projected future heat-related mortality to the key driving factors and major sources of methodological uncertainty inherent in such calculations, improving the scientific foundations of risk-based assessments of climate change and human health.

## 1. Introduction

Extreme heat events in Chicago [1,2,3], Philadelphia [4], California [5,6] and other cities and states demonstrate the potential of heat waves to cause substantial morbidity and mortality. Research has shown that exposure to extreme heat can result in increased emergency room visits and hospital admissions for a range of adverse health outcomes including cardiovascular disease, ischemic heart disease, ischemic stroke, respiratory disease, dehydration, heat stroke, diabetes, and acute renal failure [7,8,9,10,11,12,13,14,15]. Excess mortality has also been clearly linked to extreme heat [16,17,18,19,20,21,22,23]. A warming climate, with its associated increases in extreme heat events [24], therefore potentially increases the risks of these health impacts [25,26]. This paper presents a new modeling and analysis framework for estimating this changing risk that is capable of flexibly integrating diverse scenarios of future changes in key drivers, and multiple data sources for estimating health outcomes, to address a range of questions related to future heat health.

Climate and health impact studies typically apply empirically estimated temperature-mortality relationships to scenarios of present and future climate. A recent systematic review article [27] found 63 studies which provided quantitative projections of future heat-related mortality; nearly half of which (28) included one or more US cities. Most of these studies used epidemiological methods to estimate heat-related mortality relationships based on local observations of mortality and weather variables (24 of 28 studies). Of the remaining studies, three used an existing concentration-response function [28,29,30] while one [31] used concentration-response functions from five studies.

Beyond the epidemiology captured in these relationships, however, future heat-health outcomes will be determined by uncertain future trends in climate, as well as several other factors that help drive, or modulate, the health impacts of extreme heat. Many of these factors, though (along with the full range of uncertainty in all key drivers), have been insufficiently considered in research to date.

For example, an aging population increases the pool of individuals most susceptible to extreme heat events [26]. Older adults may become dehydrated and hypernatremic during hot weather, increasing the likelihood of renal failure and subsequent cardiovascular complications [32]. They may also consume medication that interferes with thermal regulation, experience preexisting medical conditions, live alone and be socially isolated, all of which increase their risk of adverse health outcomes [2,33,34,35,36]. While empirical evidence and case studies demonstrate the importance of demographics for evaluating heat-related health effects, only five of the 28 US studies reviewed in Sanderson et al. [27] included future demographic change, and only one quarter of the studies reviewed accounted for population growth at all.

In this paper, we present a new modeling and analysis framework, built around the environmental Benefits Mapping and Analysis Program—Community Edition (BenMAP, US Environmental Protection Agency, Durham, NC, USA) [37], for estimating future heat-related mortality. This new framework has the flexibility to integrate these factors within health risk assessments, as well as to provide the capability to sample across the uncertainties in these factors and drivers, to provide a more comprehensive and decision-relevant picture of the total health risk from climate-driven increases in extreme heat. We illustrate the potential of this framework by presenting an updated set of projected excess heat-related mortality estimates for the United States resulting from a range of plausible trajectories of 21st century climate change, and by assessing the sensitivity of these projections to modeling and methodological choices for: (i) climate-induced changes in future meteorological conditions; and (ii) changes in future population size and geographic distribution.

Specifically, we present estimates of heat-related mortality from BenMAP for US cities based on downscaled climate model results from the Coupled Modeling Intercomparison Project 5 (CMIP5) [38] for two Representative Concentration Pathways (RCPs) describing future greenhouse gas trajectories, RCP4.5 and RCP8.5 [39]. We use the Locating and Selecting Scenarios Online tool (LASSO, US Environmental Protection Agency, Washington, DC, USA) [40] for the first time to systematically select the specific downscaled climate realizations we use. We combine these climate modeling results with new population projections from EPA’s Integrated Climate and Land Use Scenarios (ICLUS) project [41] that have been developed to be consistent with underlying assumptions in two of the Shared Socioeconomic Pathways (SSP): SSP2 and SSP5 [42].

The subsequent sections of the paper describe in more detail these component modeling and analytic tools and scenario databases that have been integrated to develop this new assessment framework, the new heat mortality findings calculated using this framework, and next steps and potential future applications.

## 2. Materials and Methods

Climate change is increasingly being framed as a risk management problem [43,44,45], informed by core principles of risk assessment and management drawn from multiple disciplines. This framing, however, has highlighted ways in which traditional approaches to assessing climate change impacts often fail to deliver the actionable information needed by decision-makers to choose appropriate management responses for the specific climate-related risks under their purview [46,47]. Weaver et al. [43] argue for a shift in the objectives and implementation of such assessments to support risk management more concretely. They suggest improvements in three areas in particular: (i) starting with a decision focus in the design of assessments and supporting technical studies; (ii) improving quantification of the key risks most relevant to decision-makers’ needs; and (iii) presenting the resulting risk information strategically, to more effectively convey it to its end users.

We adopt such a risk-based perspective for this paper, both in the specific results we show for potential changes in heat-related mortality under a changing climate, and, more broadly, in the structure and capabilities of the new framework presented here, with a focus on the second category of recommendations from Weaver et al. [43] dealing with improved risk quantification. We address this, in part, by framing study outcomes in terms of natural metrics of “value” [48,49], in this case human mortality, and by exploring the range of scientifically plausible future outcomes under uncertainty, including lower-probability, but high-consequence futures that might have a disproportionate impact on calculations of total risk. There is substantial uncertainty surrounding many of the inputs to a climate change and health study, and for some of these uncertainty sources (e.g., the effectiveness of adaptation measures or the impact of the urban heat island effect (UHI) on heat-related mortality), there is not much research to understand processes, or it may not be clear how to appropriately represent these inputs in models. For other sources, such as changes in climate and population, there may be a wide range of scientifically supported futures. Current best practice in risk-based framing is to develop plausible scenarios of the future which capture the full, relevant range (for a given assessment, planning, or decision context) of the uncertainty in data sources, models and projections. This approach has influenced our choice of climate models and population scenarios used here to develop the new heat mortality projections presented, as described in more detail below. For each of these choices, we quantify the number and distribution of temperature-related premature deaths attributable to changes in summer-season temperature in the 2085–2095 period.

Below we describe each of the models, tools, and datasets we use in this study, focusing on recent updates and improvements. We also discuss how we selected downscaled climate model outputs using the LASSO tool, our method for quantifying temperature-related deaths using the BenMAP model, and a range of related methodological considerations and choices.

### 2.1. Population Projections

Population projections from EPA’s ICLUS project have informed previous assessments of climate change health impacts [31,50]. The ICLUS modeling framework has been revised to incorporate new and updated demographic information, and to reflect assumptions and inputs consistent with the SSPs [41]. ICLUS v2 uses county population from the 2010 Census as the starting point for projecting population. Age-specific fertility, mortality and net immigration rates specific to SSPs [51] were used in ICLUS to develop US population projections to 2100. The fourth U.S. National Climate Assessment (NCA4) [52] provided guidance for using population projections that are consistent with the risk-based approach. Following the NCA4 guidance, our analysis focuses on SSP2 and SSP5 U.S. population projections to represent our best estimate and high-population future respectively. Domestic migration was estimated with the use of a gravity model calibrated with annual Internal Revenue Service (IRS) county-to-county migration records for the years 1991–2000 [53]. Boundaries for Metropolitan and Micropolitan Statistical areas (MSAs) [54] were used to aggregate IRS migration data for both calibration and projection steps to remove high-frequency, intra-urban mobility. County-to-county migration outside of MSAs was also included in both calibration and projection steps. All components of change—fertility, mortality, international and domestic migration—were modeled on an annual time step. The resulting projections describe divergent, but plausible, distributions of population in 2090, with national totals ranging from 449 million in SSP2 to more than 678 million under SSP5. The projections are further differentiated by assumptions regarding domestic migration and international immigration, each of which affect the spatial distribution of population. Additional documentation of the ICLUS v2 model and projections is available elsewhere [41].

**Figure 1:**
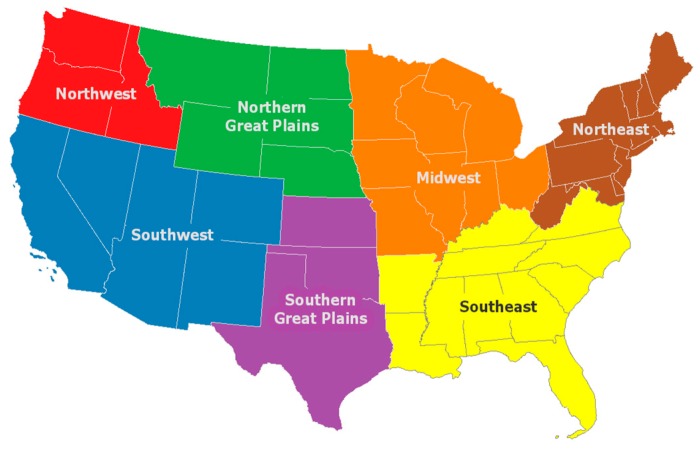
Results for this study were summarized by National Climate Assessment regions.

**Figure 2:**
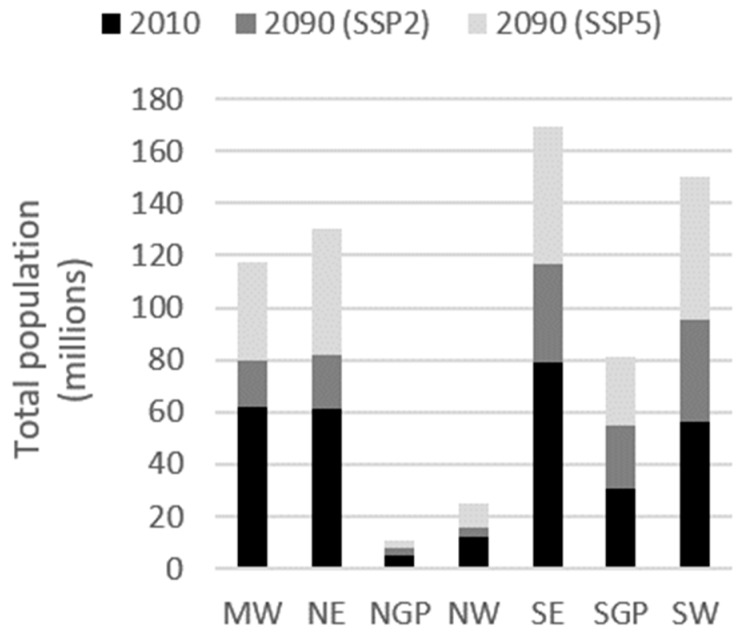
Total population by National Climate Assessment region for 2010, and for 2090 under SSP2 and SSP5 scenarios.

We also present summary results at subnational levels. We use the National Climate Assessment regions (Figure 1) to present population and heat-mortality results. Several of the regions are expected to experience substantial population growth (Figure 2). For example, the population in the Southeast is projected to increase, from 79 m people in 2010 to 116 m in 2090 for the SSP2 scenario and to over 169 m for the SSP5 scenario.

**Figure 3:**
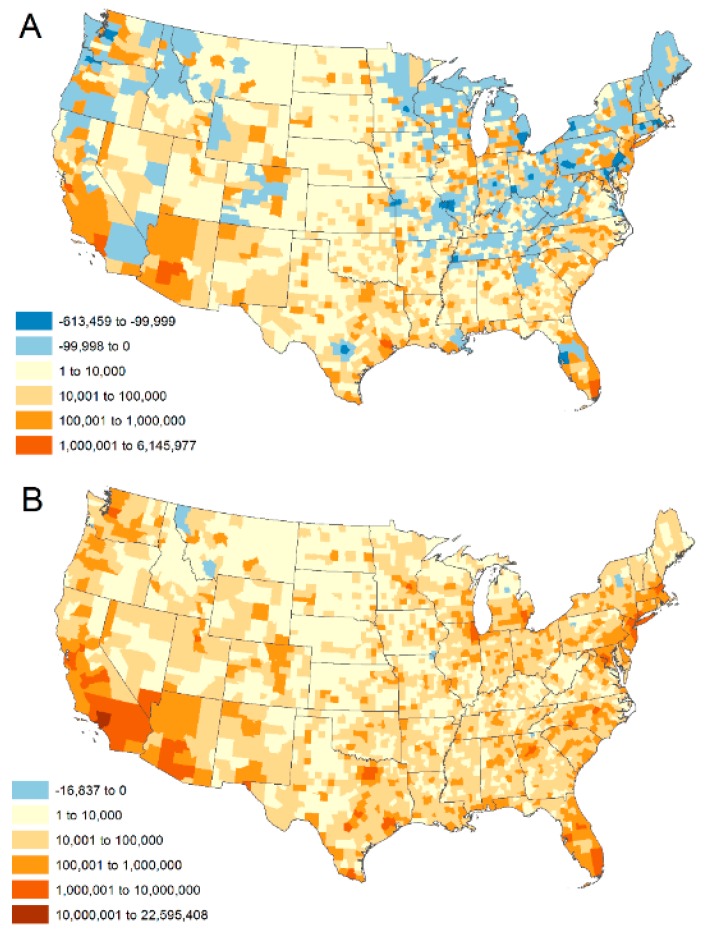
Population change by county between 2010 and 2090 under SSP2 (**A**) and SSP5 (**B**).

County-level patterns of population change between 2010 and 2090 (Figure 3) are driven by multiple assumptions used to create the projections. Relative to SSP5, the SSP2 scenario assumes lower fertility, higher mortality, and lower net immigration for all years over the projected time period. In addition, SSP2 assumes a continuation of domestic migration patterns observed during the years 1991 to 2000. The flow of net migration during this time period was generally down the urban hierarchy, that is, towards less populated areas [55,56]. For several populous metropolitan areas, these assumptions result in a decreased total population under the SSP2 scenario (Figure 3A). Conversely, natural population increase is greater under SSP5 (i.e., higher fertility, lower mortality) and net immigration is higher, relative to SSP2. Also, domestic migration flows towards Micropolitan Statistical Areas were intensified and make up a larger share of the total migrating population at each time step relative to SSP2. The combination of assumptions for SSP5 concentrates more population growth in urbanized, non-rural counties, although the large amount of population growth overall means that virtually all counties see some level of population increase by 2090 (Figure 3B).

### 2.2. Climate Change Projections

The emergence of new downscaling techniques has greatly increased the volume of climate information available for impact assessments. It is generally desirable to include a wide range of climate change projections to quantify uncertainty; logical and practical constraints frequently dictate a more parsimonious approach, however. For many studies, effectively and efficiently navigating the trade-off between rigorously addressing uncertainty and reducing redundant information is an intractable challenge. Moreover, each successive generation of climate modeling efforts and downscaling approaches has resulted in a new and unique method of data archiving, thereby adding technical sophistication to the list of challenges faced by those seeking to use the information.

In response, EPA has developed a tool that can provide some assistance in addressing these issues. LASSO [40] automates the construction of a simple heuristic device—a scatterplot—to simplify the process of identifying the most useful subset of climate projections. Similar two-dimensional approaches have appeared in previous studies [57,58,59]. For this paper, we use LASSO to select an illustrative subset of climate model outputs downscaled using the recently developed localized constructed analogs (LOCA) downscaling approach.

LOCA [60] is one of the newest statistical downscaling techniques in wide application. It was developed to address a variety of shortcomings of earlier approaches, especially preserving the daily sequence of weather events simulated by the driving General Circulation Model (GCM), essential for accurate representation of changes in extremes. LOCA has been applied to conterminous US temperature and precipitation fields from CMIP5 output for RCPs 4.5 and 8.5. These downscaled LOCA data are distributed publicly through the U.S. Bureau of Reclamation’s downscaled climate information portal [61], and serves as the primary source of climate change information for NCA4.

**Figure 4:**
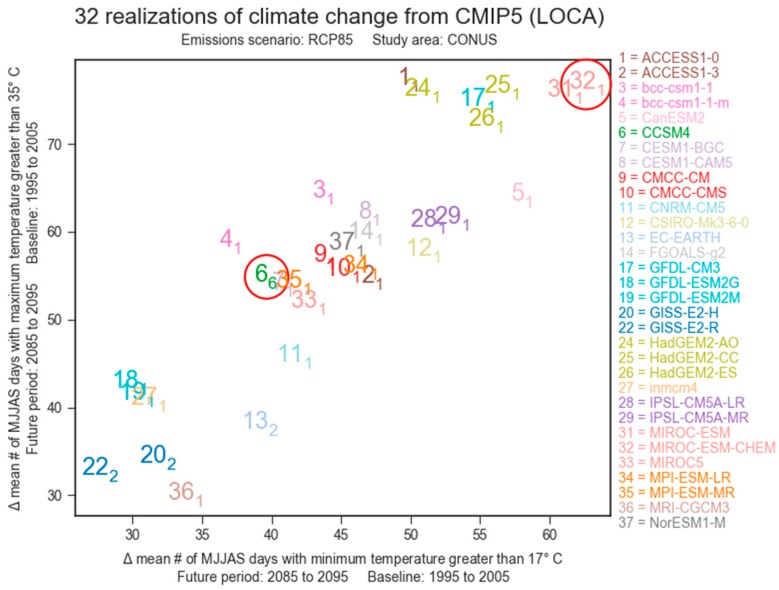
Locating and Selecting Scenarios Online (LASSO) scatterplot used to select climate projections. Climate models are denoted with a number shown to the right of the figure. Subscripts indicate the realization number of individual projections, i.e., some climate models produced multiple simulations. For this study, MIROC-ESM-CHEM (32_1_) was chosen to represent the upper bound of potential temperature change. CCSM4 (6_6_) was selected because it provided a more moderate projection of change and has been used in other recent modeling studies.

Here, we used the LASSO tool to calculate the projected change in the number of warm-season days (1 May through 30 September; “MJJAS”) in the conterminous U.S. for which the minimum temperature was greater than 17 degrees Celsius for the entire set of LOCA projections (Figure 4).

This temperature threshold represents the lower bound of the specific health impact function used for this study (see Selecting an effect coefficient below). Several of the studies reviewed by Sanderson et al. [27] used minimum or maximum temperature as the mortality variable and the health model used in this study used minimum temperature. By choosing a vertical axis for the LASSO scatterplot that describes changes in maximum daily temperature (i.e., >35 °C), we could identify projections that inform our specific research question in a way consistent with the risk framing described earlier. That is, Figure 4 allows us to identify models that project increasing daily minimums and increasing daily maximums, instead of one or the other. Although we expect daily minimum and maximum temperatures to be highly correlated, certain models—perhaps even families of models—could be considered “warmer” depending on whether daily minimums or maximums are considered. The LASSO scatterplot allows us to quickly and transparently sort climate change projections using two equally informative metrics simultaneously.

The final choice of two climate models was guided primarily by the goal of bounding our analysis with medium (CCSM4) and high-sensitivity (MIROC-ESM-CHEM) climate model projections in an efficient manner—in other words, to bracket the upper-half of the range of simulated climate change in the full ensemble of available simulations. This is consistent with a risk-based approach to climate assessment, as articulated above, which recognizes that total risk is asymmetric with respect to the distribution of future outcomes, with “worst-case” futures playing a much greater role in risk assessments than “best-case” outcomes. In the interest of brevity, we refer to these models as “CCSM” and “MIROC” throughout the rest of this paper. Although there were several potential medium-sensitivity models from which to choose, we selected CCSM because results from that climate model have been used in forthcoming air quality modeling studies. In future phases, we plan to examine whether heat and air pollution are synergistic or antagonistic when modeling combined exposures.

**Figure 5:**
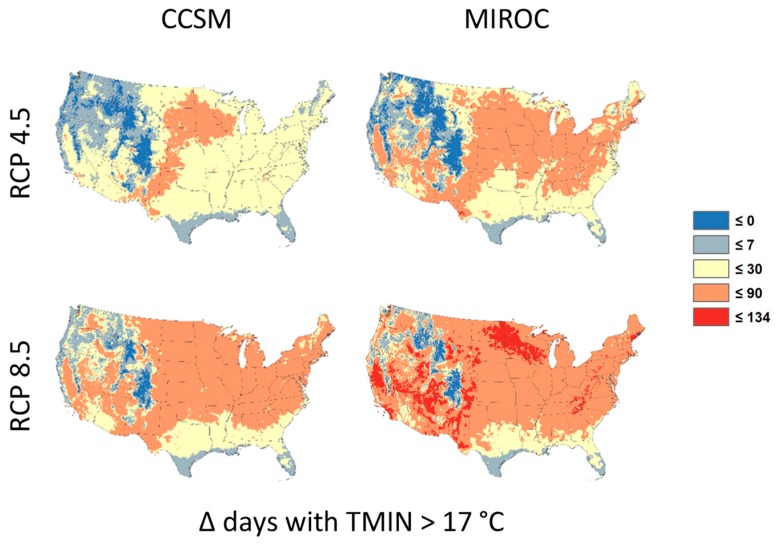
Representative maps of CCSM and MIROC climate change projections under RCP4.5 and RCP8.5 emissions scenarios. Colors denote the change in the average annualized number of warm season days where the minimum temperature exceeds 17° Celsius for the period 2085 to 2095 using 1995 to 2005 as the baseline period.

Figure 5 confirms large differences in the intensity and spatial distribution of temperature change across climate models and emissions scenarios. We limit our analysis to the May–September warm season during which virtually all heat-related health impacts would occur. As expected, the CCSM model projects an increased number of warm season days over most of the conterminous U.S. under RCP8.5 relative to RCP4.5.

Similarly, under RCP8.5 the high-sensitivity climate model, MIROC, projects relatively large increases in warm days compared to CCSM. Interestingly, coastal areas of the southeastern U.S. show small changes regardless of climate model or scenario. We note that the maximum increase in this warm season metric was 134 days, which covers nearly the entire 152-day warm season. In other words, some areas that typically experience few if any days where minimum temperature exceeds 17 °C during the warm season could exceed that threshold for nearly the entire warm season in the future. This highlights the disruptive nature of climate change and the potential for resultant human health impacts.

### 2.3. Quantifying Heat-Related Mortality

BenMAP is an open-source, PC-based tool that was designed to quantify the number and economic value of air pollution-related premature deaths and illnesses. The U.S. EPA, in collaboration with software developers from throughout the world, developed the tool to routinize and standardize the process for estimating these impacts—thus supporting air quality policies with credible estimates of the health impacts of poor air quality [37].

In this analysis, we follow the approach first detailed in Voorhees et al. [31] and substitute changes in temperature for air quality. Because Voorhees et al. applied an earlier version of the same tool, it is worth noting briefly the key differences between the two versions of the software in Table 1.

Like Voorhees et al. [37], we calculate a health impact function. A health impact function incorporates: (i) a risk coefficient from the epidemiological literature; (ii) a baseline rate of death or disease among the population of interest; (iii) a count of the number of people exposed. Temperature-related mortality is estimated by applying the health impact function with both historical and projected climate, then calculating the difference between those results. We used BenMAP to populate and process the necessary inputs and calculate the results of the health impact function.

We estimated the number of temperature-related total deaths $y_{ij}$ among adults aged 30 and above for each year i in the range 2085 to 2095 and county in the coterminous U.S. j as:

(1) $$y_{ij}=\underset{\alpha }{\sum }y_{ij\alpha }$$

(2) $$y_{ij\alpha }=m_{0ij\alpha }\cdot \left(e^{\beta \cdot C_{ij}}-1\right)\cdot P_{ij\alpha }$$

where β is the risk coefficient for all-cause mortality for adults in association with temperature, $m_{0ija}$ is the baseline all-cause mortality rate for adults aged 30 to 99 stratified in 10-year age bins, $C_{ij}$ is daily minimum temperature, and $P_{ija}$ is the number of adult residents aged 30 to 99 stratified into 5-year age bins.

We selected a temperature-mortality effect coefficient described in Medina-Ramon & Schwartz [20]. This case-crossover analysis explored the change in mortality occurring due to day-to-day changes in temperature across 50 U.S. cities between 1989 and 2000. The authors specified piecewise linear exposure variables to account for the potential J or U shape to the temperature-mortality relationship. The authors note that “… the exposure variable for heat took (a) value (of) zero when the minimum temperature was ≤17 °C …”. Thus, we imposed a threshold in the health impact function, calculating impacts only on days when the minimum temperature was at or above 17 °C. In a meta-analysis of 42 cities, the authors estimate a 5.74% (95% Confidence Interval 3.38 to 8.15) increase in the risk of death per each degree of minimum daily temperature above 17 °C. Using a national-pooled risk coefficient does not account for well-documented regional heterogeneity in the temperature-mortality relationship. However, data limitations currently prevent us from quantifying impacts using city-specific risk coefficients. The LOCA temperature surfaces (~7 km resolution) and ICLUS population projections were aggregated and disaggregated, respectively, to 12 km grid cells, which is the default spatial resolution for BenMAP. The temperature-mortality effect coefficient was applied to each 12 km grid cell, and the results aggregated by NCA region.

We applied county-level age-stratified all-cause death rates from the Centers for Disease Control and Prevention for the years 2012–2014 [62]. Using a national-level life table projected to the year 2060, provided by the U.S. Census Bureau, we projected the death rates to the year 2060. We assume that the death rates are held constant between the years 2060 and 2090.

## 3. Results

**Figure 6:**
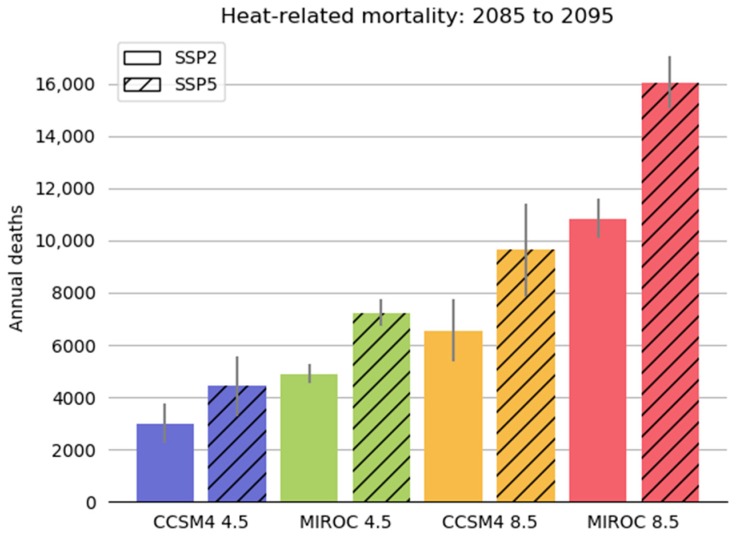
National BenMAP modeling results. The columns represent annual mortality averaged over 11 years. The thin vertical lines represent the range in annual heat mortality estimates.

Figure 6 summarizes the range of mortality estimates for the different greenhouse gas, climate model and population scenarios. The premature mortality estimates were averaged over the 11-year period (2085–2095).

For the CCSM results, the higher greenhouse gas (GHG) scenario, RCP 8.5, resulted in higher mortality estimates—9632 premature deaths for the SSP5 higher population scenario; 6545 for the SSP2 lower population scenario—compared to the lower GHG scenario RCP 4.5 (4430 to 3004 premature deaths for the SSP5 and SSP2 population scenarios respectively). The more sensitive (with respect to GHG emissions) MIROC climate model produced higher mortality estimates than the CCSM climate model, with the MIROC premature mortality estimates reaching 16,043 and 10,842 deaths for the SSP5 and SSP2 population scenarios, respectively. As expected, the lower SSP2 population scenario yielded reduced mortality estimates compared to the SSP5 population scenario, regardless of GHG scenario or climate model used.

### Regional Results

**Figure 7:**
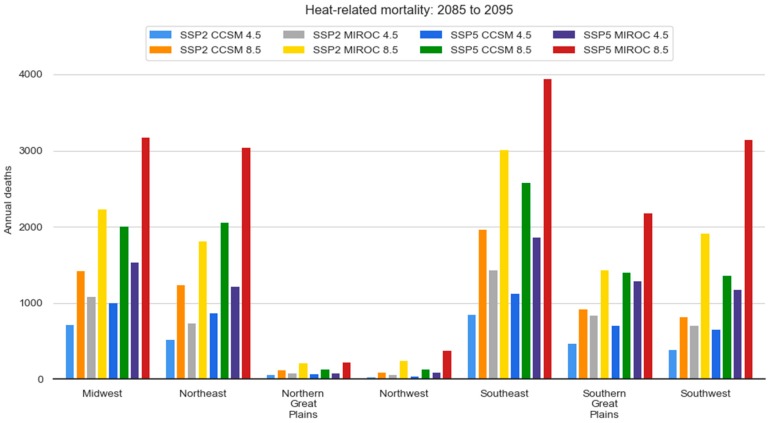
Regional modeling results by combination of population scenario, climate model, and emissions scenario.

Figure 7 shows the strong heterogeneity that we find in the regional results. The Northern Plains and the Northwest had virtually no increase in heat related mortality regardless of RCP, GCM, or population scenario. The Southeast region exhibited the greatest increase in heat-related mortality, contributing 25% or more to total US heat-related mortality across all scenarios.

Like our national estimates, the general pattern of SSP2 CCSM 4.5 resulting in the lowest heat-mortality estimates and SSP5 MIROC 8.5 resulting in the highest was observed in all regions. However, the Northeast’s SSP5 CCSM 8.5 mortality estimate (green bar) exceeded the SSP2 MIROC estimate (yellow bar; also note that they are nearly the same in the Southern Great Plains), which differs from the other regions and from the national estimates.

**Figure 8:**
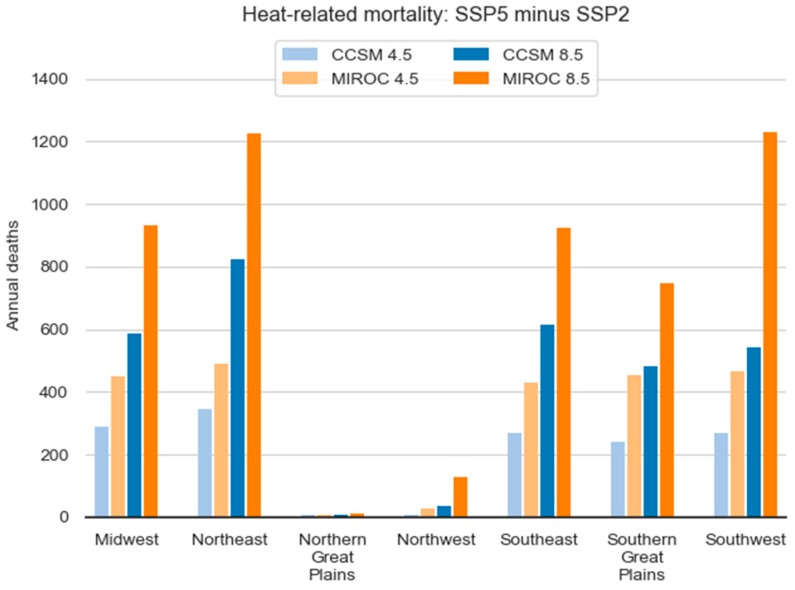
Population projections had the largest influence overall in the Northeast and Southwest.

Figure 8 illustrates the role of population in estimating heat-related mortality—a key insight that emerged from this evaluation—making clear that the choice of population projection matters greatly for projecting overall mortality. For the projections driven by the MIROC GCM, the difference between using the SSP5 and SSP2 population projection is over 1200 deaths annually in the Southwest and Northeast. The Southwest is a particularly fast-growing region under both population scenarios, and the trajectory of total population in the Northeast is substantially different depending on the population scenario. These regional population trends and patterns result from the combination of assumptions regarding immigration, domestic migration, and other demographic components used to drive ICLUS v2 projections. These results demonstrate the critical role of sub-national population projections as a tool for revealing uncertainty, generating scenarios and assessing vulnerability in the context of health impact studies.

## 4. Discussion

This study provides an updated set of projected excess heat-related mortality estimates for the United States resulting from a range of plausible trajectories of 21st century climate change (Figure 6 and Figure 7). These are new estimates, since they are based on the most recent suite of state-of-the-art emissions pathways (RCPs), GCMs (CMIP5 models), and downscaling methodologies (LOCA) as developed by the climate modeling community, and they thus represent new results compared to what has been published previously. These results show a substantial impact of projected future climate change on heat-related mortality, with many thousands of additional deaths nationally, as well as in major geographic regions of the country individually.

This study also has a larger purpose, however, in that it aims to provide a proof of concept in systematically evaluating the sensitivity of projected future heat-related mortality in the United States to the key driving factors and major sources of methodological uncertainty inherent in such calculations. The ability to carry out such systematic evaluations is ultimately aimed at improving the foundation for risk-based assessments of the human health impacts of climate change. This capability, and its implementation in practice, are urgently needed to support such risk assessments, because: (i) there are multiple sources of uncertainty with the potential to exert a first-order influence on estimates of future heat-related mortality; and (ii) there has been, to date, very little quantitative exploration of the impact of these uncertainties on published estimates.

For example, as described in the introduction, a recent systematic review of future heat-related mortality due to climate change [27] identified 28 studies that provided quantitative projections of future heat-related mortality for at least one U.S. city (out of 63 total studies globally). The authors identified several key sources of uncertainty, including:

- the spread in projections of future climate across different climate models;
- the spread in projections of future climate across different greenhouse gas scenarios;
- consideration of changes in population and demographics (and the spread across different projections of changes in these factors);
- adequate resolution of finer-scale UHI effects on top of regional climate change;
- the choice of specific mortality model used, and the way in which the mortality model was calibrated with observed data;
- if adaptation (autonomous or planned) was considered, and the specific adaptations considered.

Despite the potentially strong sensitivity of estimated mortality to these factors and methodological choices, Sanderson et al. found that very few studies analyzed their impact and explored, to any significant degree, the risk space they define. For example, only four studies used data from two or more climate models driven by the same emissions scenarios, but those studies found large differences in projected mortality based on the climate model chosen. Sanderson et al. reported similar findings for several other of the major sources of uncertainty listed above, with only a small handful of studies exploring uncertainty from changes in population and demographics, or adaptation to warmer temperatures; but with those studies demonstrating that these sources of uncertainty were of the same order of importance as the choice of climate model.

There is therefore clearly a gap which needs to be filled. This study aims to begin filling that gap by developing and applying an analytical framework designed ultimately to explore the full uncertainty space for future heat-related mortality. This parallels recent EPA efforts with respect to other health impacts (e.g., climate change and ozone, as per [50]) and impact sectors (e.g., climate change and water quality, as per [63,64].

This framework, consisting of a linked system of: (i) climate models; (ii) downscaling with LOCA; (iii) an efficient scenario selection tool; (iv) population scenarios; and (v) BenMAP, allows for systematic exploration of drivers and key uncertainties. We demonstrated the use of this framework, as described and illustrated with the mortality results above, with a preliminary evaluation of impacts associated with three of the major sources of uncertainty described in [27]:

- choice of climate model;
- choice of future greenhouse gas trajectory;
- and future changes in population size and geographic distribution.

In general, even based on this preliminary uncertainty evaluation, uncertainties associated with both future population and future climate (both as determined by GCM and emissions trajectory) exert a large influence on heat-related mortality projections—much larger than, for example, other sources of variation in these kinds of results, such as year-to-year variability in the occurrence of extreme heat events. They therefore cannot be ignored in assessments of heat-related mortality due to climate change.

**Figure 9:**
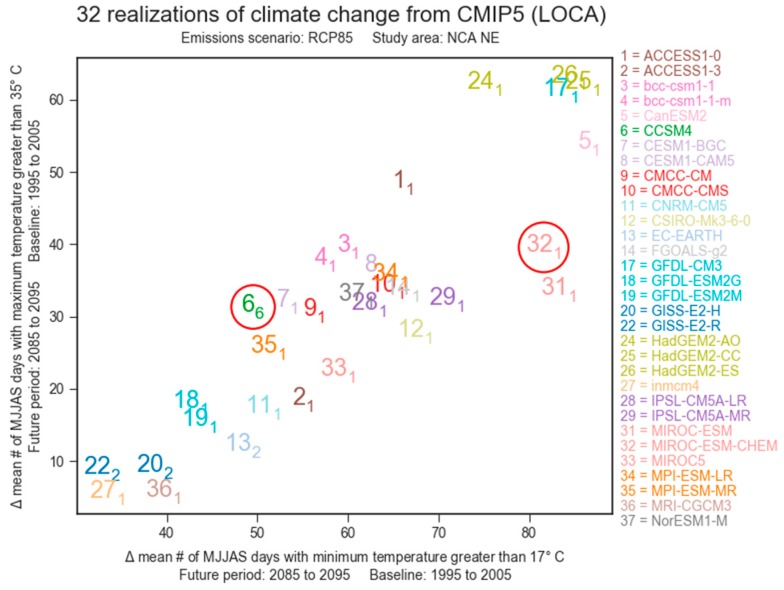
For the Northeast region, MIROC-ESM-CHEM does not fully capture the upper end of projected temperature changes.

While our analysis sought to identify a plausible upper bound of projected temperature change, consistent with the principles of risk-based framing, this effort was complicated by the necessary step of selecting a geographic boundary. Model selection in this study, while largely objective, was based on projections that were spatially averaged over the conterminous United States. The respective characterization of MIROC-ESM-CHEM and CCSM4 as high- and medium-sensitivity models holds true for most of the seven NCA regions (not shown), however alternative model selections would have been justified for the Northeast (Figure 9).

This example is notable because mortality in the Northeast was comparable to that of other regions (Figure 7), even though the warmest models for the Northeast region were not included in our analysis, indicating that the Northeast region mortality projections we present here are likely conservative. Identifying models region-by-region clearly resolves this issue, but likely increases computational burdens substantially as the volume of requisite climate information increases.

Comparing estimated changes in climate-induced changes in temperature-related mortality reported here with those reported elsewhere in the literature is complicated by critical differences in methodological choices and input parameters. This assessment applies a health impact function and uses the BenMAP tool, thus following an analytical approach that is like other studies. Voorhees [31] and colleagues quantified the number of temperature-related premature deaths occurring mid-century, estimating between about 3700 and 27,000 premature deaths, depending on the temperature-response parameter employed and the approach used to model changes in daily temperature. That paper reported temperature-attributable premature deaths quantified using the ICLUS projected population scenarios that were as much as 125% larger than the central estimates estimated using the default BenMAP projected population that did not account for changes in future climate. Post et al. [50] estimated the number of climate-induced ozone-related premature deaths occurring mid-century. Broadly consistent with our findings, they found that the estimated ozone-related deaths were sensitive to the choice of climate model and, to a lesser extent, the approach to projecting future population counts.

We reiterate that this study is largely meant to be a proof of concept, with foci of demonstrating a proposed framework and describing first-order estimates, rather than a comprehensive exploration of the full range of each critical uncertainty. As such, there are a few other factors which we did not address in our study. For example, we used only a single dose-response function in our analysis; one which produced substantially lower mortality estimates than four other functions in a previous study [31]. Our results should therefore be viewed as highly conservative, with the expectation that replicating this analysis with alternative dose-response functions would increase the estimates of heat-related mortality. The key point, however, is that BenMAP can facilitate future assessments of sensitivity across multiple dose-response functions, as well as facilitate scientific community engagement in contributing those alternative formulations to the BenMAP framework.

We also did not consider the role of the urban heat island effect (UHI) [65], which subjects urban populations to even greater heat-related health risks [35]. In fact, none of the studies in Sanderson et al. considered the impacts of urban land use changes [27]. Going forward, the use of ICLUS v2 within our integrated analytical framework can help address this, because it provides not only population and land-use projections, but also projections of impervious surface associated with urban development. This allows for an accounting of increased UHI effects resulting from population-driven land use changes and urban expansion—as well as the potential effectiveness of adaptation strategies, such as cool roofs and urban greenery [66], another set of factors that we did not consider in this analysis.

More generally, responses by individuals, public health practitioners, and local communities are key to determining the future health burdens from heat stress. There is evidence of short-term physiological acclimatization and behavioral changes [67,68] and of a long-term decreasing trend in heat-related mortality [69,70,71]. Unfortunately, the empirical evidence on heat-related adaptation does not support confident quantification of its effectiveness, nor of which specific mechanisms—behavioral, physiological, public health interventions, access to air conditioning and health care, community measures—are responsible. Gaining a better understanding of adaptation/acclimatization is critical for both estimating future heat-related mortality and for guiding efforts to reduce heat effects. The uncertainty in future heat-related mortality associated with adaptation methods may be comparable to or even larger than the uncertainty associated with emissions scenarios and climate models [29].

Finally, we could have sampled more from the uncertainties that we did consider (e.g., by using a larger suite of GCMs). Even these initial results, however, demonstrate the order of magnitude of these uncertainties and the sensitivity to them of future heat mortality estimates, as well as illustrate the potential value of the analysis framework presented here.

## 5. Conclusions

The new modeling and analytic framework presented in this paper has the potential to accelerate the development of scientific understanding regarding climate change and heat-related mortality effects. The linked system of downscaled climate model simulations, the LASSO climate scenario selection tool, the ICLUS population scenarios, and BenMAP, allows for the systematic exploration of the potential range of heat-related mortality risks and the range of uncertainties in key drivers of heat-health risks. Our results show that different combinations of methodological choices and modeling assumptions produce a wide range of projected future health impacts, particularly at regional scales, thus beginning to define the envelope of future risk with respect to greenhouse gas emission scenario, climate model, and population uncertainties. Additional simulations with the current system could further map this uncertainty space. Meanwhile, additional research on demographic change, the relationship of the UHI to heat-related mortality, how the heat-mortality relationship is changing through time and varies by geography, and the effectiveness of adaption measures is also needed. As increased knowledge of these factors becomes available, it can readily be incorporated into the framework and future assessments, a strength of our framework’s reliance on community-based models and publicly available data. Taken together, all such improvements to the scientific foundations of future heat-health risk assessment can facilitate efforts by public health departments and communities to reduce heat-related mortality.

## Figures and Tables

**Table 1:** Comparison of Benefits Mapping and Analysis Program—Community Edition (BenMAP) v4 and BenMAP-CE.

Version/Component	BenMAP v4	BenMAP-CE
Language	Delphi	C#
Source code	Proprietary	https://github.com/BenMAPCE/BenMAP-CE
Algorithm used to quantify health impacts	Health impact function	Health impact function
Database of demographic, health and economic data	Firebird SQL	Firebird SQL
Geographic information system	Tatuk (proprietary)	DotSpatial (open source)
